# Lipooligosaccharide locus classes and putative virulence genes among chicken and human *Campylobacter jejuni* isolates

**DOI:** 10.1186/s12866-016-0740-5

**Published:** 2016-11-21

**Authors:** Patrik Ellström, Ingrid Hansson, Anna Nilsson, Hilpi Rautelin, Eva Olsson Engvall

**Affiliations:** 1 Department of Medical Sciences, Clinical Microbiology, Uppsala University, SE-75185 Uppsala, Sweden; 2 Department of Medical Biochemistry and Microbiology, Zoonosis Science Center, Uppsala University, BMC A9:3, Husargatan 3, SE-75123 Uppsala, Sweden; 3 Department of Microbiology, EU Reference Laboratory for Campylobacter, National Veterinary Institute, SE-75189 Uppsala, Sweden; 4 Department of Bacteriology and Immunology, University of Helsinki, P.O. Box 21, FIN-00014 Helsinki, Finland

**Keywords:** *Campylobacter*, LOS, Pulsed Field Gel Electrophoresis (PFGE), Virulence genes, Transmission

## Abstract

**Background:**

*Campylobacter* cause morbidity and considerable economic loss due to hospitalization and post infectious sequelae such as reactive arthritis, Guillain Barré- and Miller Fischer syndromes. Such sequelae have been linked to *C. jejuni* harboring sialic acid structures in their lipooligosaccharide (LOS) layer of the cell wall. Poultry is an important source of human *Campylobacter* infections but little is known about the prevalence of sialylated *C. jejuni* isolates and the extent of transmission of such isolates to humans.

**Results:**

Genotypes of *C. jejuni* isolates from enteritis patients were compared with those of broiler chicken with pulsed-field gel electrophoresis (PFGE), to study the patterns of LOS biosynthesis genes and other virulence associated genes and to what extent these occur among *Campylobacter* genotypes found both in humans and chickens. Chicken and human isolates generally had similar distributions of the putative virulence genes and LOS locus classes studied. However, there were significant differences regarding LOS locus class of PFGE types that were overlapping between chicken and human isolates and those that were distinct to each source.

**Conclusions:**

The study highlights the prevalence of virulence associated genes among *Campylobacter* isolates from humans and chickens and suggests possible patterns of transmission between the two species.

## Background

*Campylobacter* is the most common cause of bacterial enteritis in the western world accounting for more than 200 000 reported human infections per year in the EU [1]. *Campylobacter jejuni* and *C. coli* are the two most common species infecting humans. The disease is normally self-limiting and resolves within 2–3 weeks. However, in some cases patients need to be hospitalized due to severe enteritis or to sequelae such as reactive arthritis, Guillain Barré- or Miller Fischer syndromes [2–5]. The total economic burden of campylobacteriosis has been estimated to appr. 2.4 billion euros annually in the EU [6]. *Campylobacter* is a zoonotic pathogen and the sources of infection mainly include products from farm animals and contaminated water. In many countries, poultry is the single most important source with broiler meat estimated to account for 20–30 % of human cases in the EU [6]. The prevalence of *Campylobacter* generally shows a strong seasonal variation in European countries, with a peak between June and September, both in terms of reported human cases and positive chicken flocks [1]. The mechanisms behind the pathogenicity of *C. jejuni* are poorly understood. The existence of virulence associated genes has been proposed in several studies but few have been correlated to disease severity in clinical materials [2, 4, 5, 7–10]. The structure of the lipooligosaccharide (LOS) layer of *C. jejuni* has been suggested to be an important determinant of the pathogenicity of certain postinfectious sequelae [4, 5, 10]. The synthesis of LOS in the outer membrane of *C. jejuni* is regulated by the LOS operon and based on the genomic organization, this operon has been categorized into 19 different LOS locus classes denoted A to S [11, 12]. Strains belonging to LOS locus class A, B and C harbor genes that enable sialylation of the LOS structures. Such strains have been associated with the post-infectious sequelae Guillain Barré- and Miller Fischer syndromes as well as with reactive arthritis [4, 5, 10]. Some studies have also proposed that *C. jejuni* strains with sialylated LOS might be involved in more severe symptoms of enteritis as well as invasive infection [5, 13, 14]. However in previous studies, we have not been able to find any associations between such strains and severe enteritis or with invasive infection in patients [7, 8]. The prevalence of *C. jejuni* strains with genes for sialylation of LOS among enteritis patients seems to vary between 50 and 60 % [5, 8, 15]. Although poultry is an important source of *Campylobacter* infection in many countries, very little is known about the distribution of LOS locus classes among *C. jejuni* from broiler flocks reared for consumption [15, 16]. In the present study, we compared isolates from Swedish enteritis patients with domestically acquired *C. jejuni* infection to *C. jejuni* isolates from broiler chickens raised in Sweden, with the aim to study patterns of virulence associated genes and their possible involvement in transmission from chickens to humans in Sweden.

## Results

### Species identification and PFGE typing

A total of 163 *C. jejuni* isolates were included in the study, including 104 from chicken and 59 from humans. Pulsed field gel electrophoresis (PFGE) showed that the 163 *C. jejuni* isolates fell into 66 different *Sma*I types. Of these, 19 types were unique for humans (24 isolates) and 32 types were unique for chicken (43 isolates). Fifteen *Sma*I types were detected among isolates of both human (32 isolates) and chicken (57 isolates) origin. The unique *Sma*I types each contained from 1 up to 3 human isolates or from 1 up to 4 chicken isolates. The shared *Sma*I types contained from 1 up to 7 human isolates and from 1 up to 19 chicken isolates. Seven isolates (3 from humans and 4 from chickens) were not cleaved by *Sma*I and hence, the *Sma*I type could not be determined. In total, 55 % of all isolates belonged to *Sma*I types that were shared between human and chicken isolates.

### Lipooligosaccharide (LOS) locus classes

All isolates were screened for LOS locus class by PCR. In general the distribution of LOS classes were similar among the human and chicken isolates. LOS locus class C was the most common in both materials followed by class H. The LOS locus classes with genes for incorporation of sialic acids in the LOS (classes A, B and C) together constituted 57 and 50 % of the human and chicken isolates respectively (Table 1). Temporal variations in the occurrence of the different LOS classes were analyzed but no apparent seasonal differences were observed among either human or chicken isolates.

**Table 1:** Distribution of LOS locus classes among the human and chicken *C. jejuni* isolates

LOS locus class	Human isolates	Chicken isolates
A^a^	2 (3 %)	1 (1 %)
B^a^	7 (12 %)	9 (9 %)
AB^a^	0	2 (2 %)
C^a^	25 (42 %)	40 (38 %)
E	5 (8 %)	11 (11 %)
H	12 (20 %)	17 (16 %)
18df^b^	6 (10 %)	17 (16 %)
Untypeable	2 (3 %)	13 (13 %)
Total	59	104

^a^LOS locus classes enabling sialylation of LOS

^b^Orf18df indicates isolates of the nonsialylated classes D, F, I, J, K, N, S, or Q

As expected, there was generally a strong coherence between LOS locus class and *Sma*I type where the vast majority of the isolates in one particular *Sma*I type had the same LOS locus class (data not shown). To study if certain LOS locus classes were connected to the presumed chicken source of the human isolates in this material, we analyzed the association of LOS locus class with isolates that were shared between human and chicken isolates and those that were unique to the respective source in this material. All *Sma*I types with more than 2 isolates, in total 26 (11 unique and 15 shared) were included in the comparison, covering altogether 123 isolates (44 human and 79 chicken). Among isolates with LOS locus class C there were no significant differences in the proportions belonging to the shared and the unique *Sma*I types (*P* = 0.120, Table 2). Isolates with LOS locus class H were significantly more common among *Sma*I types that were shared among chicken and humans than among *Sma*I types that were unique to each species (*P* = 0.014). LOS locus class E and H are genetically closely related [11] and an analysis of these two classes as one group showed a highly significant overrepresentation of such isolates among the shared *Sma*I types (*P* = 0.001). On the other hand, isolates with LOS locus class B were more common among *Sma*I types that were unique for chickens and humans, as compared to shared *Sma*I types (*P* = 0.0009). This was true also for the ORF18 positive isolates (*P* < 0.0001).

**Table 2:** LOS locus classes of *C. jejuni* from humans and chickens according to shared or unique *Sma*I types

LOS locus class	Shared *Sma*I types	Unique *Sma*I types	*P* value
A^a^	1	0	*1.000*
B^a^	3	7	*0.0009*
C^a^	43	7	*0.120*
E	13	1	*0.300*
H	25	1	*0.014*
E + H	38	2	*0.001*
18df^b^	7	11	*<0.0001*
Untypeable	4	0	*0.575*
Total	96	27	

^a^LOS locus classes enabling sialylation of LOS

^b^Orf18df indicates isolates of the nonsialylated classes D, F, I, J, K, N, S, or Q

### Putative virulence genes

The prevalences of the putative virulence genes were generally similar between the human and chicken isolates (Table 3). The gene *fucP* was detected in 64 % of the human- and 55 % of the chicken isolates respectively (*P* = 0.232), whereas *virB*11 was not found at all among the human isolates and only in 11 % of the tested chicken isolates. The genes *ceuE, pldA* and *ciaB* were present in the vast majority of both human and chicken isolates. We analyzed if the presence of these genes in the *C. jejuni* isolates was related to season but no significant seasonal variation was found. The largest difference was found between *fucP* positive vs *fucP* negative among human isolates in June (*P* = 0.281). As for the LOS locus classes, there was strong coherence between the putative virulence genes and *Sma*I type (data not shown). Furthermore, the presence of the gene *fucP* was strongly associated with certain LOS locus classes, both in the human and chicken isolates (Table 4). In particular, isolates of LOS locus class C were almost exclusively *fucP* positive, whereas nearly all isolates of LOS locus classes E and H were *fucP* negative. Isolates with LOS locus classes A and B were generally *fucP* positive.

**Table 3:** Distribution of the putative virulence genes studied among human and chicken *C. jejuni* isolates

Gene	Human isolates	Chicken isolates
*fucP*	38 (64 %)	57 (55 %)
*ceuE*	58 (98 %)	104 (100 %)
*pldA*	58 (98 %)	39/45 (87 %)^a^
*ciaB*	59 (100 %)	45/45 (100 %)^a^
*virB11*	0 (0 %)	5/45 (11 %)^a^
Total	59	104

^a^Only 45 of the 104 chicken isolates were tested for the presence of these genes

**Table 4:** Human and chicken isolates of *C. jejuni* according to LOS locus class and presence of the gene *fucP*

LOS locus class	*fucP* positive	*fucP* negative	*P* value
A^a^	3	0	*NS*
B^a^	12	4	*NS*
C^a^	62	3	*P < 0.0001*
E	0	16	*P < 0.0001*
H	2	27	*P < 0.0001*
Total	95	68	

^a^LOS locus classes enabling sialylation of LOS

## Discussion

In this study on human and chicken *C. jejuni* isolates obtained during year 2009 and analyzed by PFGE, we found that the majority of isolates (55 %) had *Sma*I types that were shared between human and chicken isolates. Assuming that this is a result of transmission between the two species, it supports the current notion that chicken is a major source of human *C. jejuni* infections. Interestingly, we found that the number of *Sma*I types that were unique either to chickens (*n* = 32) or to humans (*n* = 19) were higher than the number of *Sma*I types that were shared between chickens and humans (*n* = 15). This could suggest that there are only certain genotypes among the Swedish chicken isolates that are more likely to be transmitted to humans.

When studying the distribution of LOS locus classes as well as the presence of the putative virulence genes *fucP*, *ceuE, virB*11, *pldA* and *ciaB* in the human and chicken isolates it was found that the LOS locus classes showed similar distributions among the human and chicken isolates. The LOS locus class C was the most common in both sources followed by the classes E and H, which is in line with the results from other studies on human isolates [5, 8, 15]. By a combined analysis of LOS locus classes and the *Sma*I restriction patterns, we sought to identify potential differences in transmission of isolates with different LOS locus classes between the Swedish chickens and humans as well as from unknown sources. As the chicken isolates in this study represented approximately 99 % of all chickens that were produced in Sweden during 2009, and the human isolates represent all patients in a specific region that have sought medical care for gastrointestinal symptoms attributed to domestically acquired *C. jejuni* infection, it could be assumed that a human *C. jejuni* isolate with a *Sma*I pattern identical to a chicken isolate most likely actually originated from this chicken source. Human or chicken isolates with *Sma*I patterns that were unique to either source on the other hand, could be assumed to originate from other sources including environmental, other farm animals or imported chicken. With this analysis we found that isolates of LOS locus class C were equally represented among isolates with *Sma*I types that were shared between the Swedish chickens and humans as among those that were unique, suggesting that such isolates are readily transmitted between the Swedish chickens and humans, but were also acquired from other sources. Isolates of LOS locus class E and H were significantly more likely to have shared *Sma*I patterns than unique ones, suggesting that such isolates are to a high extent spread between Swedish chickens and humans. Isolates with LOS locus class B on the other hand showed an inversed distribution with significantly more isolates having *Sma*I patterns unique to either chickens or humans, suggesting that such isolates were rarely spread between the two species in this material. The same was true for the ORF 18 positive isolates although this group does not represent a single LOS locus class.

In line with the results from previous studies, we found that nearly all isolates, both from humans and chickens had the gene *ceuE*, suggesting that this gene is important both for human infection and colonization of chickens. The ability of *C. jejuni* isolates to utilize L-fucose, mediated in part by the fucose permease gene *fucP,* has been suggested to provide an advantage in the colonization of chickens and piglets [17, 18]. In this material however, the prevalence of *fucP* was only slightly higher than 50 % among the human and chicken isolates. In our previous studies we found this particular gene to be present in 49 % of isolates from enteritis patients and in 23 % of isolates from bacteremia patients in Finland [2, 8]. Together these results suggest that L-fucose utilization by *fucP* hardly is a prerequisite for colonization of chickens or for causing infections in humans.

## Conclusions

In conclusion, we found that the prevalences of the different LOS locus classes in general, and of the putative virulence factors *fucP*, *ceuE*, *pldA* and *ciaB* were similar among human and chicken *C. jejuni* isolates in Sweden. However, LOS locus classes E and H were more abundant among isolates with *Sma*I types that were shared between Swedish chickens and humans whereas LOS locus class B was more abundant among isolates with *Sma*I types unique to the sources. Altogether, these results suggest that humans might acquire *C. jejuni* isolates with different LOS locus classes from different sources. Taking into account the association between *C. jejuni* isolates with some LOS locus classes and post infectious sequelae such as Guillain Barré syndrome and reactive arthritis in humans, this is an interesting finding that needs further studies in larger materials.

## Methods

### Bacterial isolates

Human isolates of domestic origin (as determined from the travel history of the patients) during 2009 (*N* = 60) were obtained at the Clinical Microbiology Laboratory at Uppsala University Hospital. Chicken isolates were collected within the frame of the Swedish *Campylobacter* Monitoring Programme for broilers. This has been run by the Swedish Poultry Meat Association since 1991 [19]. In the programme, which includes 99 % of broilers in Sweden, samples were taken from every broiler flock at slaughter. Ten caecal samples were pooled into one composite sample from each flock. In 2009, more than 3000 chicken flocks were sampled and approximately 12 % were positive for *Campylobacter*. One isolate from every second positive chicken flock was selected for the study, making up a total of 110 isolates from 52 different broiler producers. Preliminary species identification was performed by microscopic examination of morphology and motility as well as oxidase, catalase and hippurate test [20, 21].

All isolates were then identified to species level by PCR [22–24]. For this, DNA was extracted using the Qiagen blood and tissue kit (Qiagen, Sollentuna, Sweden) according to the manufacturer´s instructions. The following primer pairs were used: MDmapA1 upper- and MDmapA2 lower primers targeting the *mapA* gene of *C. jejuni* (annealing temperature (AT) =59 °C) [22] as well as the COL3 upper- and MDCOL2 lower primers targeting the *ceuE* gene of *C. coli* (AT = 59 °C) [22, 23]. Primer pair: C412F and C1228R (AT = 58 °C) targeting a region of the 16S rRNA gene specific for the genus *Campylobacter* was used to confirm successful DNA extraction [24]. All primers were purchased from Eurogentec (Ougrée, Belgium). Cycling conditions were 10 min at 95 °C, 25 cycles of 30s at 95 °C, 90s at annealing temp. and 1 min at 72 °C, with a final extension step of 10 min at 72 °C. The strains *C. jejuni* NCTC11168 (ATCC 700819), *C. jejuni* 81176 (ATCC BAA-2151) and *C. coli* LMG6440 were used as positive controls and omission of template was used as negative control. Seven isolates (1 human and 6 chicken) were determined as *C. coli* and excluded from the study.

### Genotyping of *C. jejuni* by Pulsed field Gel Electrophoresis (PFGE)

All isolates were analyzed by PFGE according to standard protocols. The restriction enzyme *Sma*I was used for cleavage of whole genomic DNA obtained from *C. jejuni* cultured on blood agar plates. For electrophoresis the instrument CHEF- DR II (BioRad, Stockholm, Sweden) was used. The BioNumerics program version 7.5 (Applied Maths, Kortrijk, Belgium) was used for analysis of restriction patterns of the isolates. Isolates with identical restriction patterns were designated as belonging to the same *Sma*I type.

### Putative virulence associated genes

All *C. jejuni* isolates were analyzed by PCR for the presence of genes encoding the putative virulence factors, fucose permease (*fucP*) and the iron transport protein (*ceuE*). Furthermore all human isolates and 45 chicken isolates were screened for presence of the genes phospholipase (*pldA*), the *Campylobacter* invasion antigen (*ciaB*) and the plasmid borne virulence gene (*virB11*). A close to 100 % prevalence of the genes *pldA* and *ciaB* as well as very low prevalence of *virB11* were expected among the chicken isolates from previous studies, and hence only a subset was selected to verify this [25]. The reaction mixture was 1x AmpliTaq Gold 360 buffer with 1.25U of AmpliTaq Gold 360 polymerase (Applied Biosystems, Austin, USA), 200 μM dNTP (Fermentas, St. Leon-Rot, Germany), 0.2 μM of each primer (Eurogentec, Ougrée, Belgium) and 5 μl of template DNA in a total volume of 25 μl. Cycling conditions were 95 °C for 10 min followed by 25 cycles of 95 °C for 30s, annealing temperatures (*fucP*: 58 °C, *ceuE*: 60 °C, *pldA*: 45 °C, *ciaB*: 58 °C, *virB11*: 53 °C) for 30s and 72 °C for 60s. For *virB11* a touch down protocol was run with 5 cycles at 53 °C, 5 cycles at 52 °C and 15 cycles at 51 °C. All reactions ended with an extension step at 72 °C for 7 min. *C. jejuni* NCTC 11168 and *C. jejuni* 81176 were used as positive controls and omission of template was used as negative control.

### Determination of LOS locus class

LOS locus class was determined for all isolates by PCR screening as described by Parker et al. [11, 12]. Primer names and sequences were as described in [12]. Amplification of the open reading frame (orf) 12 (*waaV*) was used to verify successful DNA extraction. The LOS locus classes A and B were identified by the presence of orf7ab (*cstII*), orf6ab1 (*cgtB-1*), orf6ab2 (*cgtB-2*) and orf5bII (cgtA2). Class C was identified by the presence of orf6c (*cgtB*) and orf7c (*cstIII*). The classes E, H, O and P were identified by the presence of orf26e and orf27e. The orf18df, present in isolates of the nonsialylated classes D, F, I, J, K, N, S, or Q was used for isolates that were untypeable with the primers above. Cycling conditions were 95 °C for 10 min followed by 30 cycles of 30s at 94 °C, 30s at 52 °C, and 60s at 72 °C and final extension at 72 °C for 7 min. Primers for orf6ab1 and orf6ab2 were run together in a duplex PCR with an annealing temperature of 59 °C.

### Statistical analysis

Statistical analyses were performed with GraphPad Prism version 5 (GraphPad Software, San Diego, CA, USA) The χ^2^ or Fisher’s exact tests were used for comparison of categorical data. A *P* value < 0.05 was considered significant.

## Abbreviations

AT, Annealing temperature; *ceuE*, Gene encoding an iron transport protein; *cgtB*, β-1,3-galactosyltransferase; *ciaB*, *Campylobacter* invasion antigen; *cstII, cstIII*, Sialyltransferases; *fucP*, Fucose permease; LOS, Lipooligosaccharide; ORF, Open reading frame; PFGE, Pulsed field gel electrophoresis; *pldA*, phospholipase gene; *SmaI*, Restriction enzyme from *Serratia marcescens*; U, Units; *virB11*, Plasmid borne virulence gene; *waaV*, Putative glycosyltransferase
